# Clinical experiences and predictors of success of treatment with vedolizumab in IBD patients: a cohort study

**DOI:** 10.1186/s12876-021-01604-z

**Published:** 2021-01-22

**Authors:** Laura Mühl, Emily Becker, Tanja M. Müller, Raja Atreya, Imke Atreya, Markus F. Neurath, Sebastian Zundler

**Affiliations:** Department of Medicine 1 and “Deutsches Zentrum Immuntherapie”, University Hospital Erlangen, Friedrich-Alexander-Universität Erlangen-Nürnberg, Ulmenweg 18, 91054 Erlangen, Germany

**Keywords:** Inflammatory bowel diseases, Vedolizumab, Real-world data

## Abstract

**Background:**

Vedolizumab has become a standard treatment for the inflammatory bowel diseases ulcerative colitis (UC) and Crohn’s disease (CD). However, there is an ongoing debate on the ideal individual treatment algorithms and means to predict treatment response are not routinely established.

**Aims:**

We aimed to describe our experiences with vedolizumab at a large German tertiary referral center and to identify clinical predictors of success of vedolizumab treatment.

**Methods:**

We performed a retrospective single-center cohort study employing univariable and multivariable analyses as well as Kaplan–Meier analyses of persistence on treatment.

**Results:**

36% and 35% of the patients with UC and CD, respectively, reached clinical remission after 17 weeks. Patients with lower clinical disease activity were more likely to achieve remission. The median persistence on treatment was 33 months for UC and 29 months for CD.

**Conclusion:**

Our study confirms that vedolizumab is an efficient option for the treatment of UC and CD. Clinical parameters of disease activity may help to predict the success of treatment.

## Background

The choice of treatment options in inflammatory bowel diseases (IBD) is constantly growing [1]. However, due to primary non-response or secondary loss of response in a relevant part of the patients [2–5], many of them do still not achieve durable remission and suffer from disease symptoms and complications.

While the increasing number of therapeutic agents provides the opportunity to initiate further therapy lines with alternative compounds in these patients, more and more evidence indicates that the probability of treatment success steeply declines with each line of treatment [6]. This observation underscores the necessity to choose the right treatment for individual patients early on to optimize their long-term outcomes and to reduce healthcare costs [7]. However, head-to-head comparative studies on the efficacy of the available therapies are scarce [8] and, moreover, biomarkers to predict the individual probability of treatment success have not been routinely established so far. Thus, evidence supporting individualized treatment strategies is largely lacking and preferences of the prescriber and the patient play a relevant role in therapy selection [9].

One of the standard therapy options for the treatment of both Crohn’s disease (CD) and ulcerative colitis (UC) is the anti-α4β7 integrin antibody vedolizumab. Its efficacy and safety had been shown in large phase III trials in 2013 [3, 10] and confirmed in multiple real-world reports since then [11–14]. Vedolizumab has been shown to prevent the so-called gut homing process of α4β7-expressing immune cells, i.e. their extravasation from the blood to the intestinal tissue [15–17]. Probably due to this impact on circulating and not on resident immune cells, the onset of the effect is somewhat delayed in a considerable portion of the responding patients [10]. Therefore, vedolizumab is seen as a rather “slow-acting” antibody [18], further emphasizing the importance of a high a priori likelihood of success to avoid long ineffective treatment periods before the response or non-response can be reliably assessed.

While several potential biomarkers have been reported [19–21], such approaches require further validations before entering clinical practice. To the contrary, clinical characteristics of patients are easy to collect and may directly help to estimate outcomes. Thus, using clinical parameters for such purposes has been previously suggested [22].

Here, we retrospectively analyzed a cohort of patients treated with vedolizumab at a German tertiary referral center and performed an exploratory investigation of the ability of several routinely available parameters to predict the success of treatment. Overall, our data confirm that vedolizumab is an efficient option for the treatment of UC and CD and suggest that patients with less severe disease are more likely to enter remission. Thus, they further validate previous findings, while also providing important new aspects supporting the concept that treatment decisions for or against vedolizumab might be based on clinical patient characteristics.

## Methods

### Study design

We performed a single-center retrospective cohort study. All patients having received vedolizumab at the Department of Medicine 1 of the University Hospital Erlangen between June 2014 and May 2020 were identified. The individual patient data were collected from electronic patient records, anonymized and analyzed in accordance with the approval by the institution’s Ethics Committee (Ethics Committee of the Friedrich-Alexander-University Erlangen-Nuremberg; 288_20Bc).

### Inclusion and exclusion criteria

IBD patients aged ≥18 years and receiving at least three applications of vedolizumab were screened for eligibility. Exclusion criteria were previous proctocolectomy, microscopic colitis, IBD-undefined (IBD-U), an active entero- or colostoma during the follow-up and an incomplete follow-up.

### Baseline parameters and outcomes

Baseline was defined as the date of the first application of vedolizumab. The following baseline parameters were collected: age at disease onset, duration of disease, clinical disease activity as assessed by the 9-point partial Mayo score (PMS) [23] and the Harvey–Bradshaw-Index (HBI) [24] for UC and CD, respectively, disease extent (E and L category of the Montreal classification for UC and CD, respectively), B category of the Montreal classification for CD patients, previous anti-TNF-α exposure, previous bowel surgery, current smoking status, endoscopic activity as assessed with the endoscopic Mayo score for UC and the simplified endoscopic score for CD (within 3 months prior to baseline), corticosteroid treatment of the first flare, concurrent steroid treatment and extra-intestinal manifestations at baseline as well as the laboratory parameters C-reactive protein (CRP), hemoglobin (Hb) and leukocytes.

The primary endpoint of this study was clinical remission at the time of the fifth application of vedolizumab (17 ± 0.5 weeks). Clinical remission was defined as active treatment and a PMS  ≤ 1  for UC or an HBI  ≤ 4 for CD. As secondary endpoints, we analyzed corticosteroid-free clinical remission, i.e. a PMS  ≤ 1 or an HBI  ≤  4 for UC and CD, respectively, without concomitant steroid treatment at the time of the fifth application of vedolizumab, clinical remission and corticosteroid-free clinical remission for UC and CD after 1 year of treatment and endoscopic remission for UC defined as an endoscopic Mayo score ≤ 1 at follow-up endoscopy (mean follow-up of 8.9 ± 0.7 months).

Additionally, we recorded, whether the patients stayed on treatment over a maximum follow-up of 3 years.

### Statistical analysis

GraphPad Prism (GraphPad Software, Inc.) was used to perform statistical analyses.

Categorical variables are reported as frequencies and continuous variables as mean values with standard error of the mean (SEM) or range as indicated. In the univariable analyses, categorical variables were compared using Fisher’s exact test (for frequencies < 5 and contingency tables > 2 × 2) or Chi-square test (other cases) Continuous variables were compared using Mann–Whitney-U test for nonparametric data or unpaired t-test for parametric data.

In a second step, we built multivariable logistic regression prediction models for the UC and the CD cohort. Baseline clinical parameters with a p-value < 0.2 on univariable analysis were included [25] after assessment for collinearity. The ability of the models to identify patients achieving the endpoints was analyzed with receiver-operator characteristic (ROC) analysis.

Persistence on treatment in our cohort during 3 years after therapy initiation was visualized by calculation of “survival” probabilities with the Kaplan–Meier method. Comparisons were performed with the log-rank test.

Significance levels are indicated by asterisks (*p < 0.05; **p < 0.01; ***p < 0.001).

## Results

### Patient demographics and efficacy of vedolizumab

**Fig. 1:**
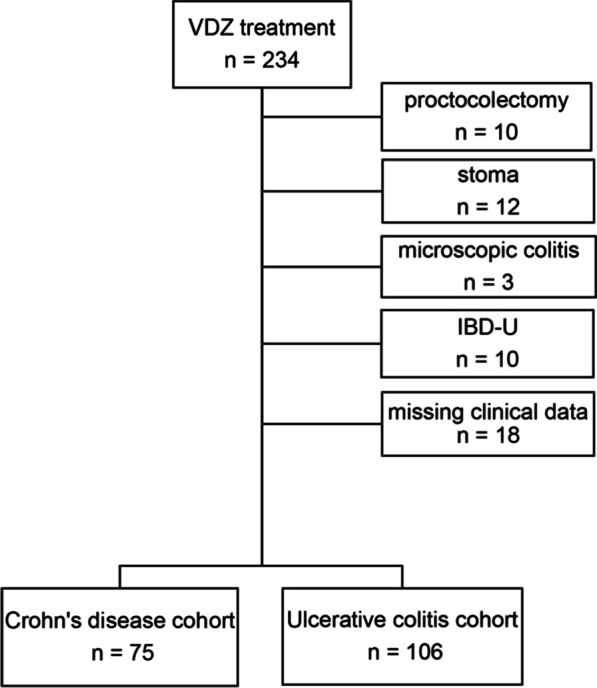
Patient inclusion and exclusion chart. From 234 eligible patients 53 had to be excluded as indicated on the right. 106 patients with UC and 75 patients with CD were included into the analyses

We identified 234 IBD patients that received vedolizumab at the Department of Medicine 1 of the University Hospital Erlangen from June 2014 to May 2020. After exclusion of 53 patients due to previous proctocolectomy (n = 10), IBD-U (n = 10), microscopic colitis (n = 3), active entero- or colostoma (n = 12) or incomplete follow-up (n = 18), 181 patients were included into the analysis, 106 with UC and 75 with CD (Fig. 1).

Baseline characteristics of the patient cohort are summarized in Table 1. Most patients received intensified treatment (mean application interval from application 3 to 5 was 4.8 weeks for UC and 4.7 weeks for CD) due to lack of response or primary application of an escalated regimen.

**Table 1:** Baseline characteristics of patients with Crohn’s disease (CD) and Ulcerative colitis (UC)

	Patients with CD	Patients with UC
Disease duration from initial diagnosis (years)	12.9 (0.5–44.3)	8.3 (0.1–51.3)
Age at initial diagnosis (years)	25.5 (10.5–66.8)	34 (4.0–77.3)
Female (%)	47/75 (63)	46/106 (43)
CRP (mg/L)	11 (0.2–78.3)	8.5 (0.2–62.8)
Hb (g/dL)	12.9 (9.1–16.5)	12.7 (7.5–16.8)
Leukocytes × 10^3^/µL	9.9 (3.6–20)	9.3 (2.4–22.5)
Endoscopic disease activity*	13.3 (0–26)	2.3 (0–3)
Clinical disease activity**	7.03 (0–22)	4 (0–8)
Disease extent (%)***	L1: 15/75 (20)	E1: 7/104 (7)
	L2: 10/75 (13)	E2: 51/104 (49)
	L3: 50/75 (67)	E3: 46/104 (44)
First flare treated with corticosteroids (%)	63/73 (86)	86/102 (84)
Previous treatment with anti TNF-α antibody (%)	67/75 (89)	79/105 (75)
Number of previous anti TNF-α antibodies (%)	1: 14/67 (21)	1: 43/79 (54)
	2: 52/67 (78)	2: 31/79 (39)
	3: 1/67 (1)	3: 5/79 (6)
Active smoking (%)	21/75 (28)	8/105 (8)
Concomitant corticosteroid therapy (%)	42/74 (57)	53/101 (52)
Concomitant immunosuppressant use (%)	Azathioprin: 3/74 (4)	Azathioprin: 6/102 (6)
	6-Mercaptopurin: 0/74 (0)	6-Mercaptopurin: 2/102 (2)
	MTX: 5/74 (7)	MTX: 0/102 (0)
Prior bowel surgery (%)	22/75 (29)	1/106 (1)
CD phenotype*** (%)	B1: 18/75 (24)	
	B2: 30/75 (40)	
	B3: 27/75 (36)	
Extraintestinal manifestations (%)	25/75 (33)	14/106 (13)

Data are presented as mean (minimal value—maximal value) or frequencies (%)

^*^Endoscopic Mayo score for UC, simplified endoscopic score for Crohn’s disease (SES-CD) for CD

^**^PMS (partial Mayo score) for UC, HBI (Harvey Bradshaw-Index) for CD

^***^According to the Montreal classification

36% of the patients with UC and 35% of the patients with CD met the primary endpoint of clinical remission at week 17 ± 0.5 and short-term steroid-free clinical remission was achieved by 29% of the patients with UC and CD. 71% and 64% of the patients with UC and CD, respectively, which received continued vedolizumab therapy and for which data were available achieved clinical remission after 1 year. Long-term corticosteroid-free clinical remission was achieved by 68% and 56% of the patients. 45% of the patients with UC had endoscopic remission during follow up (mean 8.9 ± 0.7 months) (Table 2).

**Table 2:** Efficacy of vedolizumab

	Patients with clinical remission (%)		Patients with corticosteroid-free remission (%)		Patients with endoscopic remission (%)
	Week 17 ± 0.5	1 year	Week 17 ± 0.5	1 year	Month 8.9 ± 0.7
UC	38/106 (36)	40/56 (71)	31/106 (29)	38/56 (68)	29/64 (45)
CD	26/75 (35)	16/25 (64)	22/75 (29)	14/25 (56)	

### Baseline clinical disease activity and hemoglobin levels are associated with induction of remission in UC

We evaluated the association of the collected baseline parameters with success of therapy as assessed by the endpoints clinical remission, steroid-free clinical remission and endoscopic remission in univariable analyses.

In patients with UC, baseline Hb levels were significantly higher in patients achieving clinical remission than in patients not achieving clinical remission. Moreover, baseline clinical disease activity as assessed by the 9-point PMS was significantly lower in patients that entered clinical remission compared with those who did not (Table 3). Similarly, with regard to steroid-free clinical remission, Hb was higher in remitters than in non-remitters, while the difference in PMS shortly missed significance (Additional file 1: Table 1). Moreover, lower PMS scores were significantly associated with clinical remission (Additional file 1: Table 2) and almost significantly associated with steroid-free clinical remission (Additional file 1: Table 3) after 1 year of treatment. Likewise, it was significantly lower in patients achieving vs. not achieving endoscopic remission during follow-up (Additional file 1: Table 4).

**Table 3:** Clinical remission at week 17 ± 0.5: univariable analysis of baseline parameters in patients with UC

	UC patients with clinical remission	UC patients with no clinical remission	p-value	
Disease duration from initial diagnosis (years)	10.0 ± 1.4	7.4 ± 1.1	0.09	^M^
Age at initial diagnosis (years)	35.7 ± 2.4	32.7 ± 1.8	0.31	^M^
CRP (mg/L)	10.5 ± 2.4	7.6 ± 1.4	0.36	^M^
Hb (g/dL)	13.3 ± 0.4	12.4 ± 0.3	0.04	^M^
Leukocytes × 10^3^/µL	9.3 ± 0.5	9.4 ± 0.5	0.82	^M^
Endoscopic disease activity	2.3 ± 0.2	2.3 ± 0.1	0.99	^M^
Clinical disease activity (PMS)	3.5 ± 0.3	4.4 ± 0.2	0.02	^M^
Disease extent (E1/E2/E3)/n (%)	(2/22/14)/38 (5/58/37)	(5/28/31)/64 (8/44/48)	0.38	^C^
First flare treated with corticosteroids (%)	34/37 (92)	50/63 (79)	0.16	^F^
Previous treatment with anti TNF-α antibody (%)	27/38 (71)	50/65 (77)	0.64	^F^
Active smoking (%)	2/37 (5)	6/66 (9)	0.71	^F^
Concomitant corticosteroid therapy (%)	20/37 (54)	32/61 (53)	> 0.99	^F^
Prior bowel surgery (%)	0/38 (0)	1/66 (2)	> 0.99	^F^
Extraintestinal manifestations (%)	5/38 (13)	9/68 (13)	> 0.99	^F^

Data are presented as mean ± standard error of mean or frequencies (%)

^M^Mann–Whitney-U test

^C^Chi-square test

^F^Fisher exact test

We incorporated all parameters with a p-value < 0.2 into multivariable logistic regression models. Here, clinical disease activity at baseline emerged as independent predictor for short-term clinical remission (Table 4), while both Hb and PMS independently predicted short-term steroid-free clinical remission and PMS independently predicted endoscopic remission. No parameter reached significance with regard to long-term clinical outcomes (Additional file 1: Table 5).

**Table 4:** Multivariable logistic regression analysis in patients with UC

Parameter	OR	95% CI	p-value	
Clinical remission at week 17 ± 0.5				
Disease duration from initial diagnosis	1	0.1 to 1.01	0.11	ns
Hb [g/dL]	1.27	1.00 to 1.66	0.06	ns
Clinical disease activity	0.71	0.52 to 0.95	0.03	*
First flare treated with corticosteroids	3.38	0.82 to 18.4	0.12	ns

*OR* odds ratio

**Fig. 2:**
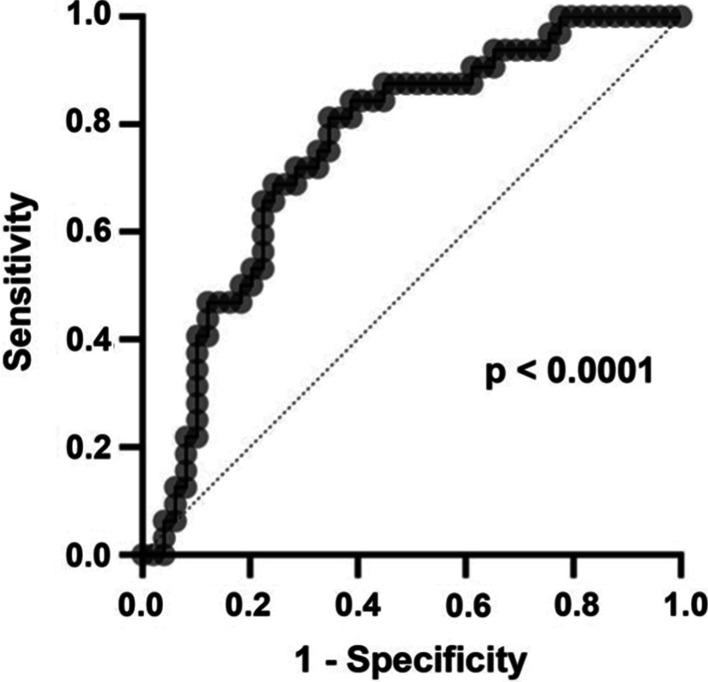
Receiver–operator characteristic (ROC) analysis of the performance of the multivariable model to predict clinical remission in patients with UC

In a ROC analysis of the multivariable model to predict short-term clinical remission, our model achieved an area under the curve (AUC) of 0.76 with a negative predictive power of 71.7% and a positive predictive power of 71.4% (Fig. 2).

### Baseline clinical disease activity predicts induction of clinical remission in CD

Similarly, we performed univariable analyses to identify associations between the clinical baseline parameters and clinical remission or steroid-free remission in patients with CD.

Consistent with the observations for UC, baseline clinical disease activity measured with the HBI was significantly lower in patients entering remission early during vedolizumab treatment than in patients with persisting clinical disease activity. Moreover, previous anti-TNF-α antagonist exposure was higher in patients with non-remission (Table 5). Both parameters were also significantly different between patients achieving short-term steroid-free clinical remission and no steroid-free clinical remission. Here, additionally and not surprisingly, concomitant steroid treatment at baseline was substantially higher in those not achieving steroid-free remission (Additional file 1: Table 6). Hb levels closely missed significance regarding both endpoints and were numerically higher in patients entering remission and steroid-free remission. While no significant associations could be identified for clinical remission after 1 year (Additional file 1: Table 7), patients achieving corticosteroid-free clinical remission after 1 year were, on average, younger and had a longer disease duration (Additional file 1: Table 8).

**Table 5:** Clinical remission at week 17 ± 0.5: univariable analysis of baseline parameters in patients with CD

	CD patients with clinical remission	CD patients with no clinical remission	p-value	
Disease duration from initial diagnosis (years)	11.8 ± 1.9	13.4 ± 1.3	0.42	^M^
Age at initial diagnosis (years)	28.8 ± 2.8	23.4 ± 1.5	0.09	^M^
CRP (mg/L)	7.0 ± 1.4	13.5 ± 3.1	0.43	^M^
Hb (g/dL)	13.5 ± 0.4	12.6 ± 0.3	0.05	^U^
Leukocytes × 10^3^/µL	8.9 ± 0.7	10.4 ± 0.7	0.16	^M^
Endoscopic disease activity	14.4 ± 1.4	12.9 ± 1.5	0.58	^U^
Clinical disease activity (HBI)	5.9 ± 0.8	9.4 ± 0.8	0.002	^M^
Disease extent (L1/L2/L3)/n (%)	(3/5/18)/26 (12/19/69)	(11/5/31)/47 (23/11/66)	0.34	^C^
First flare treated with corticosteroids (%)	18/24 (75)	43/47 (92)	0.08	^F^
Previous treatment with anti TNF-α antibody (%)	20/26 (77)	46/47 (98)	0.007	^F^
Active smoking (%)	5/26 (19)	15/47 (32)	0.29	^F^
Concomitant corticosteroid therapy (%)	12/26 (46)	28/44 (64)	0.21	^F^
Prior bowel surgery (%)	7/26 (27)	15/47 (32)	0.79	^F^
Phenotype (B1/B2/B3)/n (%)	(7/11/8)/26 (27/42/31)	(11/18/18)/47 (24/38/38)	0.81	^C^
Extraintestinal manifestations (%)	7/26 (27)	18/49 (37)	0.45	^F^

Data are presented as mean ± standard error of mean or frequencies (%)

^M^Mann–Whitney-U test

^U^Unpaired t test

^C^Chi-square test

^F^Fisher exact test

Again, we built a multivariable logistic regression model. As in UC, baseline clinical disease activity independently predicted short-term clinical remission (Table 6). The achievement of short-term steroid-free clinical remission was independently associated with previous anti-TNF-α exposure and the age at initial diagnosis, while no parameter significantly associated with outcomes after 1 year (Additional file 1: Table 9).

**Table 6:** Multivariable logistic regression analysis in patients with CD

Parameter	OR	95% CI	p-value	
Clinical remission at week 17 ± 0.5				
Age at initial diagnosis (years)	1.06	0.99–1.13	0.09	ns
Hb (g/dl)	1.30	0.79–2.23	0.31	ns
Leukocytes × 10^3^/ul	1.00	0.81–1.24	0.98	ns
HBI	0.82	0.68–0.96	0.03	*
First flare treated with corticosteroids	0.29	0.04–2.02	0.22	ns
Previous treatment with anti TNF-α antibody	0.06	0.002–1.09	0.08	ns

*OR* odds ratio

**Fig. 3:**
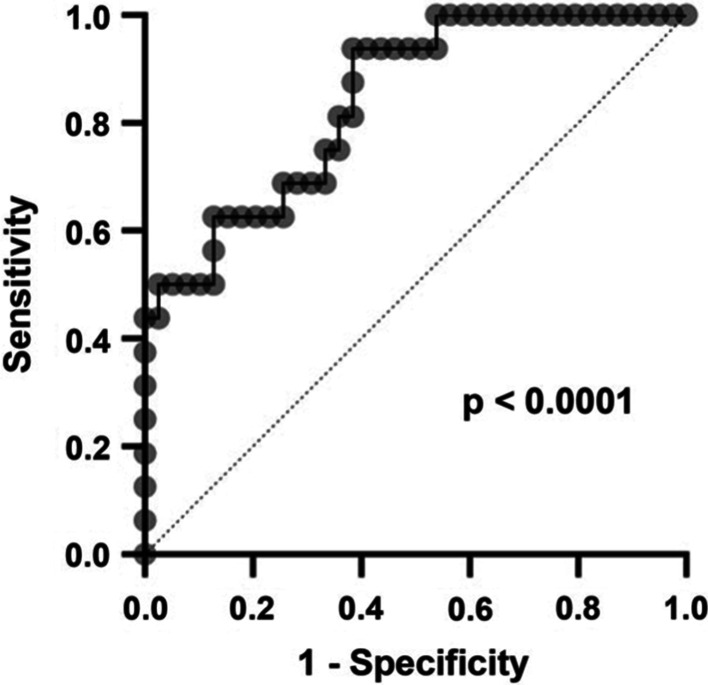
Receiver–operator characteristic (ROC) analysis of the performance of the multivariable model to predict clinical remission in patients with CD

The AUC of our multivariable model to predict short-term clinical remission in CD in a ROC analysis was 0.84. The negative and positive predictive power were 81.4% and 66.7%, respectively (Fig. 3).

### Persistence on treatment with vedolizumab

We also analyzed, for how long patients in our cohort continued to receive vedolizumab over a maximum follow-up of 3 years.

**Fig. 4:**
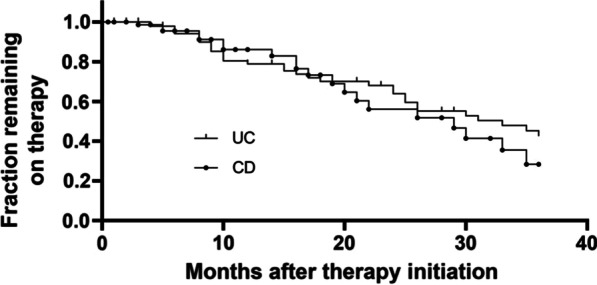
Kaplan–Meier graph showing the persistence of patients with UC and CD on vedolizumab treatment

After 12 months, 78.9% of the patients with UC and 86.1% of the patients with CD further received vedolizumab treatment. After 24 months, this was the case in 63.9% and 56.1% of the UC and CD patients, respectively. And after 36 months, 42.6% of the UC patients and 28.4% of the CD patients persisted on treatment (Fig. 4). The median duration of therapy was 33 months for UC and 29 months for CD.

## Discussion

Phase III clinical trials pose strict criteria for the inclusion and exclusion of patients resulting in treatment cohorts that do not always adequately reflect the patients later actually treated with the drugs investigated [26]. Hence, real-world reports are an important tool to assess, whether the data of the pivotal clinical studies translate to broader and less well selected populations. Several studies reporting real-world experiences with vedolizumab have been published so far [12, 27–29]. Although the endpoints employed in these studies slightly differed, our data fit into the overall picture and the efficacy we observed is comparable to previous reports.

We report a median persistence on therapy of 33 and 29 months for UC and CD, respectively. This is comparable to what has been observed for first-line anti-TNF-α antibodies [30] and is remarkable, since a majority of our cohort was previously exposed to one or more biological therapies.

Prediction of the success of treatment is one of the major unmet needs in the field of IBD. With the sole exception of the recent head-to-head trial of vedolizumab vs. adalimumab in UC [8], there are no high-quality comparative data available that provide guidance for therapeutic decisions. Hence, evidence on the best choice of treatments and their optimal sequential use are largely lacking [31].

While further head-to-head studies would be suitable to address this aspect, it is not realistic that appropriate studies will be performed in near future to consistently answer this question in heterogeneous patient populations. Thus, alternative strategies to decide, which agent to use in which patient are needed.

Accordingly, several “biomarker strategies” have been suggested, e.g. assessment of intestinal anti-TNF-α target cells by confocal laser endoscopy to predict response to adalimumab [32] or quantification of baseline αE expression in the gut to predict response to etrolizumab [33, 34]. Such approaches have also been investigated with regard to vedolizumab. E.g., baseline microbial signatures were associated with the success of treatment [20], while transcriptomic signatures failed to predict outcomes [35]. Our group had observed that dynamic adhesion of peripheral blood CD4^+^ T cells to MAdCAM-1 in vitro and early changes in the expression of α4β1 integrin on circulating CD4^+^ T cells correlate with clinical response [21, 36, 37].

However, those strategies will require substantial additional efforts before entering clinical practice and will, therefore, not be available in the near future. Consistently, predicting treatment success based on clinical patient characteristics has previously been suggested as an alternative [38, 39] and several studies have reported clinical parameters associating with different endpoints of treatment with vedolizumab. Stallmach et al. reported the absence of prior anti-TNF-α treatment and minimal steroid therapy before the initiation of vedolizumab as predictors of clinical remission of UC at week 54 [29]. In a US real-world report, CRP levels > / = 8.0 mg/L were negatively associated with clinical response and remission at week 14 [40]. Moreover, mild clinical disease activity at baseline predicted clinical remission of CD at week 14 in an Israeli multicenter cohort [13]. A US consortium reported prior anti-TNF-α exposure as negative predictor of remission in both CD and UC and of response in UC. Additionally, active or historical smoking, severe baseline disease activity and active perineal disease made clinical remission of CD less likely [28, 41]. Chaparro et al. showed that mild disease activity of UC was associated with higher probability of clinical remission of UC at week 14, whereas high CRP resulted in lower probability. Similarly, higher baseline HBI scores reduced the probability of clinical remission of CD at week 14 [42]. This was also resembling the observations of Baumgart et al., who reported low HBI scores and the absence of hospitalization in the year prior to treatment initiation to predict clinical remission of CD at week 14 [43]. And in the French GETAID cohort, corticosteroid use at induction and an HBI score > 10 were negative predictors of steroid-free clinical remission of CD at week 14 and 54, while a Mayo score > 9 at induction was a negative predictor of steroid-free clinical remission of UC at week 14 and 54 [11, 27]. Altogether, these reports strengthen the concept that patients with milder disease activity are more likely to benefit from therapy with vedolizumab.

Our study confirms and endorses these observations, since, consistently, lower HBI or PMS scores were associated with short-term clinical remission and steroid-free clinical remission in UC and CD and long-term clinical remission and endoscopic remission in UC. It is evident that our study has some important limitations, such as retrospective single-center design potentially implying bias to our data as well as limiting the data basis for long-term analyses, and unavailability of a validation cohort for the multivariable models. However, it contributes another important piece to the puzzle and helps to draw a clearer picture of the clinical use of vedolizumab. A previously not reported aspect we observed is that Hb, a broadly available parameter, which is probably reflecting rectal bleeding and, thus, mucosal integrity in this scenario, seems to be a parameter helping to assess the probability of achieving remission.

Both for CD and UC, clinical decision support tools deriving from similar analyses in the phase III studies and validated in other real-world populations have previously been published [39, 44, 45]. Although it is clear that they rely on data of higher quality, a potential disadvantage of those models is that they require information on endoscopic disease activity. Our prediction models had acceptable AUCs purely based on clinical parameters. However, validation in independent populations is required.

Of note, increased response rates in patients with milder disease activity are not an exclusive feature of vedolizumab, but have also been observed with other antibodies like ustekinumab [38]. This leads to the question, how far predictors identified in this and other studies are able to actually support treatment decisions by weighing the balance towards one or the other treatment option or whether they are just a surrogate of a more or less refractory disease course [46]. The answer to this question is still open. This will require further and broader prospective studies including patients treated with different therapeutic options. For the moment, clinicians need to rely on gradual differences in the predictive clinical parameters as well as taking into account safety aspects, extraintestinal manifestations or the route of administration.

## Conclusion

In our cohorts of UC and CD patients, the efficacy of vedolizumab in inducing clinical and steroid-free remission was comparable to previous reports. Median persistence on therapy was almost 3 years. Lower clinical disease activity was associated with improved outcomes. Taken together, our data may help to better understand the role of vedolizumab in treatment algorithms and to support treatment decisions.

## Supplementary information

**Additional file 1**. Univariable and multivariable analyses for secondary endpoints.

## Data Availability

The datasets generated and analyzed during the current study are available from the corresponding author on reasonable request.

## Abbreviations

AUC: Area under the curve
BSA: Bovine serum albumin
CD: Crohn’s disease
CRP: C-reactive protein
HBI: Harvey–Bradshaw index
IBD: Inflammatory bowel disease
MAdCAM-1: Mucosal addressin vascular cell adhesion molecule 1:
PBMCs: Peripheral blood mononuclear cells
PMS: Partial Mayo score
rh: Recombinant human
ROC: Receiver operator characteristics
SEM: Standard error of the mean
TNF-α: Tumor necrosis factor alpha
UC: Ulcerative colitis
VDZ: Vedolizumab
