# Supplementary material for: Clinical experiences and predictors of success of treatment with vedolizumab in IBD patients: a cohort study
Source: BMC Gastroenterol. 2021 Jan 22;21:33. doi: 10.1186/s12876-021-01604-z (PMC7821503; doi:10.1186/s12876-021-01604-z)
Supplement: Supplementary file 1 — Additional file 1. Univariable and multivariable analyses for secondary endpoints. [file 12876_2021_1604_MOESM1_ESM.pdf]

Supplementary Table 1

Corticosteroid-Free Remission at week 17+/- 0.5: Univariable analysis of baseline parameters in patients with UC.

	UC patients with CLINICAL STEROID-FREE REMISSION	UC patients with NO CLINICAL STEROID-FREE REMISSION	p-value	
Disease duration from initial diagnosis [years]	9.2 ± 1.6	8.0 ± 1.0	0.59	M
Age at initial diagnosis [years]	35.5 ± 2.5	33.0 ± 1.8	0.38	M
CRP [mg/L]	10.1 ± 2.8	7.9 ± 1.3	0.81	M
Hb [g/dL]	13.4 ± 0.4	12.4 ± 0.3	0.03	M
Leukocytes x10³/µL	9.5 ± 0.7	9.3 ± 0.5	0.54	M
Endoscopic disease activity	2.3 ± 0.2	2.2 ± 0.1	0.52	M
Clinical disease activity	3.5 ± 0.3	4.2 ± 0.2	0.05	M
Disease extent (E1/E2/E3)/n (%)	(2/18/11)/31 (6/58/36)	(5/33/34)/72 (7/46/47)	0.51	C
First flare treated with corticosteroids (%)	28/30 (93)	57/71 (80)	0.14	F
Previous treatment with anti TNF-α antibody (%)	22/31 (71)	56/73 (77)	0.62	F
Active smoking (%)	2/30 (7)	6/74 (8)	>0.99	F
Concomitant corticosteroid therapy (%)	14/30 (47)	38/69 (55)	0.51	F
Prior bowel surgery (%)	0/31 (0)	1/74 (1)	>0.99	F
Extraintestinal manifestations (%)	4/31 (13)	10/75 (13)	>0.99	F

Data are presented as mean ± standard error of mean or frequencies (%).

[M] Mann-Whitney-U test

[C] Chi-square test

[F] Fisher exact test

[U] Unpaired t test

Supplementary Table 2

Clinical remission after one year of treatment: Univariable analysis of baseline parameters in patients with UC.

	UC patients with CLINICAL REMISSION after one year on treatment	UC patients with NO CLINICAL REMISSION after one year on treatment	P value	
Disease duration from initial diagnosis [years]	10.3±1.8	9±2.05	0.85	M
Age at initial diagnosis [years]	32.7±2.29	36.9±3.74	0.35	M
CRP [mg/L]	2.22±9.09	1.28±6.41	0.68	M
Hb [g/dL]	13.3±0.38	12.8±0.45	0.23	M
Leukocytes x10³/µL	9.54±0.70	9.04±0.83	0.66	M
Endoscopic disease activity	2.32±0.15	2.23±0.23	0.74	M
Clinical disease activity	0.26±3.5	0.47±4.6	0.049	M
Disease extent (E1/E2/E3)/n (%)	(4/17/18)/39 (10/44/46)	(1/10/5)/16 (6/63/31)	0.44	C
First flare treated with corticosteroids (%)	34/39 (87)	11/15 (73)	0.24	F
Previous treatment with anti TNF-α antibody (%)	27/39 (69)	14/16 (88)	0.19	F
Active smoking (%)	2/40 (5)	1/16 (6)	>0.99	F
Concomitant corticosteroid therapy (%)	21/39 (54)	10/16 (63)	0.77	F
Extraintestinal Manifestations (%)	2/40 (5)	3/16 (19)	0.14	F

Data are presented as mean ± standard error of mean or frequencies (%).

[M] Mann-Withney-U test

[C] Chi-square test

[F] Fisher exact test

Supplementary Table 3

Clinical corticosteroid-free remission after one year of treatment: Univariable analysis of baseline parameters in patients with UC.

	UC patients with CLINICAL STEROID-FREE REMISSION after one year on treatment	UC patients with NO CLINICAL STEROID-FREE REMISSION after one year on treatment	P value	
Disease duration from initial diagnosis [years]	10.6±1.86	8.75±1.83	0.87	M
Age at initial diagnosis [years]	31.6±2.26	38.7±3.58	0.11	M
CRP [mg/L]	9.03±2.33	6.85±1.34	0.52	M
Hb [g/dL]	13.2±0.4	13±0.43	0.48	M
Leukocytes x10³/µL	9.55±0.75	9.06±0.74	0.77	M
Endoscopic disease activity	2.39±0.15	2.13±0.22	0.32	M
Clinical disease activity	3.5±0.26	4.47±0.44	0.08	M
Disease extent (E1/E2/E3)/n (%)	(4/16/17)/37 (11/43/46)	(1/11/6)/18 (6/61/33)	0.45	C
First flare treated with corticosteroids (%)	32/37 (86)	13/17 (76)	0.44	F
Previous treatment with anti TNF-α antibody (%)	26/37 (70)	15/18 (83)	0.35	F
Active smoking (%)	2/38 (5)	1/18 (6)	>0.99	F
Concomitant corticosteroid therapy (%)	19/37 (51)	12/18 (67)	0.39	F
Extraintestinal Manifestations (%)	2/38 (5)	3/18 (17)	0.31	F

Data are presented as mean ± standard error of mean or frequencies (%).

M Mann-Withney-U test

C Chi-square test

F Fisher exact test

Supplementary Table 4

Endoscopic remission: Univariable analysis of baseline parameters in patients with UC.

	UC patients with ENDOSCOPIC REMISSION	UC patients with NO ENDOSCOPIC REMISSION	P value	
Disease duration from initial diagnosis [years]	10.1±2.01	7.83±1.3	0.54	M
Age at initial diagnosis [years]	33.4±2.83	32.4±2.49	0.73	M
CRP [mg/L]	9.34±2.51	8.45±1.65	0.84	M
Hb [g/dL]	12.7±0.45	12.2±0.39	0.39	U
Leukocytes x10³/µL	10.1±0.73	9.27±0.63	0.42	M
Endoscopic disease activity	2.41±0.15	2.29±0.11	0.28	M
Clinical disease activity	3.79±0.31	4.88±0.32	0.02	M
Disease extent (E1/E2/E3)/n (%)	(3/17/9)/29 (10/52/59)	(2/15/17)/34 (6/44/50)	0.30	C
First flare treated with corticosteroids (%)	22/27 (81)	28/33 (85)	0.74	F
Previous treatment with anti TNF-α antibody (%)	24/29 (83)	28/34 (82)	>0.99	F
Active smoking (%)	2/29 (7)	3/35 (9)	>0.99	F
Concomitant corticosteroid therapy (%)	14/27 (52)	30/42 (71)	0.13	F
Extraintestinal Manifestations (%)	6/29 (21)	3/35 (9)	0.28	F

M Mann-Withney-U test

U Unpaired t test

C Chi-square test

F Fisher exact test

Supplementary Table 5

Multivariable logistic regression analysis of secondary endpoints in patients with UC.

PARAMETER	OR	95% CI (profile likelihood)	P value	P value summary
CINICAL CORTICOSTEROID-FREE REMISSION at week 17+/-0.5				
Hb [g/dL]	1.32	1.02 to 1.76	0.04	*
Clinical disease activity	0.73	0.52 to 0.98	0.04	*
First flare treated with corticosteroids	4.13	0.89 to 30.9	0.10	ns
CLINICAL REMISSION after one year				
Endoscopic disease activity	2.07	0.69 to 6.57	0.19	ns
Clinical disease activity	0.63	0.37 to 0.99	0.06	ns
Previous treatment with anti TNF-α antibody	0.27	0.03 to 1.81	0.21	ns
Extraintestinal Manifestations	0.28	0.02 to 2.97	0.27	ns
CINICAL CORTICOSTEROID-FREE REMISSION after one year				
Age at initial diagnosis [years]	0.97	0.92 to 1.01	0.09	ns
Clinical disease activity	0.69	0.46 to 1	0.06	ns
ENDOSCOPIC REMISSION				
Clinical disease activity	0.71	0.51 to 0.98	0.04	*
Concomitant corticosteroid therapy	0.49	0.15 to 1.47	0.21	ns

OR, Odds ratio

Supplementary Table 6

Corticosteroid-free remission at week 17+/-0.5: Univariable analysis of baseline parameters in patients with CD.

	CD patients with CLINICAL STEROID-FREE REMISSION	CD patients with NO CLINICAL STEROID-FREE REMISSION	p-value	
Disease duration from initial diagnosis [years]	13.1 ± 2.1	12.8 ± 1.3	0.90	M
Age at initial diagnosis [years]	28.4 ± 2.6	24.0 ± 1.6	0.08	M
CRP [mg/L]	6.1 ± 1.6	13.3 ± 2.8	0.12	M
Hb [g/dL]	13.6 ± 0.4	12.6 ± 0.3	0.06	U
Leukocytes $x10^3/\mu L$	8.5 ± 0.7	10.5 ± 0.6	0.08	M
Endoscopic disease activity	14.9 ± 1.7	12.9 ± 1.4	0.46	U
Clinical disease activity	6.3 ± 0.9	8.9 ± 0.7	0.04	M
Disease extent (L1/L2/L3)/n (%)	(2/3/17)22 (9/14/77)	(12/7/32)/51 (24/14/63)	0.34	C
First flare treated with corticosteroids (%)	15/20 (75)	46/51 (90)	0.13	F
Previous treatment with anti TNF-α antibody (%)	17/22 (77)	49/51 (96)	0.02	F
Active smoking (%)	4/22 (18)	16/51 (31)	0.39	F
Concomitant corticosteroid therapy (%)	8/22 (36)	32/48 (67)	0.02	F
Prior bowel surgery (%)	6/22 (27)	16/51 (31)	0.79	F
Phenotype (B1/B2/B3)/n (%)	(5/11/6)/22 (23/50/27)	(13/18/20)/51 (26/35/39)	0.47	C
Extraintestinal manifestations (%)	7/22 (32)	18/53 (34)	0.795	F

Data are presented as mean ± standard error of mean or frequencies (%).

[M] Mann-Withney-U test

[U] Unpaired t test

[C] Chi-square test

[F] Fisher exact test

Supplementary Table 7

Clinical remission after one year of treatment: Univariable analysis of baseline parameters in patients with CD.

	CD patients with CLINICAL REMISSION after one year on treatment	CD patients with NO CLINICAL REMISSION after one year on treatment	P value	
Disease duration from initial diagnosis [years]	16.6±2.44	11±2.15	0.19	M
Age at initial diagnosis [years]	25.6±2.94	30.3±3.13	0.19	M
CRP [mg/L]	4.95±1.11	9.16±2.77	0.12	U
Hb [g/dL]	14.2±0.53	12.8±0.56	0.10	U
Leukocytes x10³/µL	11.8±1.23	11±1.51	0.68	U
Endoscopic disease activity	13±1.93	14.4±4.08	0.75	U
Clinical disease activity	8.33±1.47	10±2.1	0.63	M
Disease extent (L1/L2/L3)/n (%)	(1/3/12)/16 (6/19/75)	(4/1/4)/9 (44/11/44)	0.07	C
First flare treated with corticosteroids (%)	12/15 (80)	7/8 (88)	>0.99	F
Previous treatment with anti TNF-α antibody (%)	14/16 (88)	9/9 (100)	0.52	F
Active smoking (%)	5/16 (31)	1/8 (13)	0.62	F
Concomitant corticosteroid therapy (%)	7/15 (47)	4/9 (44)	>0.99	F
Phenotype (B1/B2/B3)/n (%)	(5/8/3)/16 (31/50/19)	(3/2/4)/9 (33/22/44)	0.29	C
Extraintestinal Manifestations (%)	7/16 (44)	2/9 (22)	0.40	F

[M] Mann-Withney-U test

[U] Unpaired t test

[C] Chi-square test

[F] Fisher exact test

Supplementary Table 8

Clinical corticosteroid-free remission after one year of treatment: Univariable analysis of baseline parameters in patients with CD.

	CD patients with CLINICAL STEROID-FREE REMISSION after one year on treatment	CD patients with NO CLINICAL STEROID-FREE REMISSION after one year on treatment	P value	
Disease duration from initial diagnosis [years]	17.8±2.63	10.41±1.78	0.03	M
Age at initial diagnosis [years]	23.5±2.64	32.1±3.28	0.04	M
CRP [mg/L]	4.26±1.11	9.08±2.24	0.06	U
Hb [g/dL]	14±0.65	13.2±0.52	0.39	U
Leukocytes x10³/µL	11.7±1.55	11.2±1.20	0.84	M
Endoscopic disease activity	11.8±1.86	15.2±3.42	0.44	U
Clinical disease activity	7.08±1.33	11.2±1.93	0.17	M
Disease extent (L1/L2/L3)/n (%)	(1/3/10)/14 (7/21/71)	(4/1/6)/11 (36/9/55)	0.18	C
First flare treated with corticosteroids (%)	10/13 (77)	9/10 (90)	0.60	F
Previous treatment with anti TNF-α antibody (%)	12/14 (86)	11/11 (100)	0.49	F
Active smoking (%)	3/14 (21)	4/11 (36)	0.66	F
Concomitant corticosteroid therapy (%)	6/13 (46)	5/11 (46)	>0.99	F
Phenotype (B1/B2/B3)/n (%)	(5/7/2)/14 (36/50/14)	(3/3/5)/11 (27/27/46)	0.22	C
Extraintestinal Manifestations (%)	7/14 (50)	2/11 (18)	0.21	F

[M] Mann-Withney-U test
[U] Unpaired t test
[C] Chi-square test
[F] Fisher exact test

Supplementary Table 9

Multivariable logistic regression analysis of secondary endpoints in patients with CD.

PARAMETER	OR	95% CI (profile likelihood)	P value	P value summary
CINICAL CORTICOSTEROID-FREE REMISSION at week 17+/-0.5				
Age at initial diagnosis [years]	1.1	1.02 to 1.2	0.02	*
CRP [mg/dL]	1.01	0.9 to 1.08	0.82	ns
Hb [g/dl]	1.50	0.81 to 3.12	0.22	ns
Leukocytes x10³/ul	1.02	0.75 to 1.35	0.90	ns
HBI	0.9	0.74 to 1.06	0.22	ns
First flare treated with corticosteroids	0.66	0.08 to 6.23	0.70	ns
Previous treatment with anti TNF-α antibody	0.03	0.001 to 0.62	0.04	*
Concomitant corticosteroid therapy	0.52	0.08 to 3.41	0.56	ns
CLINICAL REMISSION after one year				
Disease Duration from initial diagnosis	1.02	1.00 to 1.06	0.09	ns
Age at initial diagnosis [years]	0.87	0.66 to 1.03	0.17	ns
CRP [mg/dL]	1.57	0.93 to 4.09	0.19	ns
Hb [g/dl]	11.9	1.70 to 17	0.1	ns
Disease Extent	2.05	0.21 to 26.4	0.52	ns
CINICAL CORTICOSTEROID-FREE REMISSION after one year				
Disease Duration from initial diagnosis	1.01	0.1 to 1.04	0.16	ns
Age at initial diagnosis [years]	0.88	0.7 to 1.03	0.17	ns
CRP [mg/dL]	0.95	0.64 to 1.35	0.79	ns
HBI	0.94	0.75 to 1.16	0.57	ns
Disease Extent	1.72	0.42 to 9.72	0.47	ns

OR, Odds ratio
